# Co-expression of candidate genes regulating growth performance and carcass traits of Barki lambs in Egypt

**DOI:** 10.1007/s11250-022-03263-y

**Published:** 2022-08-11

**Authors:** Nasser Ghanem, Mohamed Zayed, Ismail Mohamed, Mona Mohammady, M. F. Shehata

**Affiliations:** 1 Department of Animal Production, Faculty of Agriculture, Cairo University, El-Gamaa Street, Giza, 12613 Egypt; 2 Faculty of Agriculture, Cairo University Research Park, Cairo University, Cairo, Egypt; 3 Department of Animal and Poultry Breeding, Animal and Poultry Division, Desert Research Center, Cairo, Egypt

**Keywords:** Barki lambs, Growth rate, Carcass traits, Gene expression

## Abstract

Sheep are considered one of the main sources of animal protein in Egypt and the producers of sheep mutton eagers to find biological criteria for selecting fast-growing lambs that reach market weight early. Therefore, the present study aimed to find a link between the expression profile of selected candidate genes with growth performance and carcass traits of Barki lambs. Thirty-eight Barki lambs were kept and fed individually after weaning till 12 months of age and were divided into 3 groups according to growth performance (fast, intermediate, and slow-growing). Three samples were taken from different body tissues (eye muscle, liver, and fat tail) of each group, directly during slaughtering and stored at − 80 °C until RNA isolation. Real-time PCR was used to profile selected candidate genes (RPL7, CTP1, FABP4, ADIPOQ, and CAPN3) and GAPDH was used as a housekeeping gene. The results indicated that the final body weight was significantly (*P* ≤ 0.05) greater in the fast (49.9 kg) and intermediate (40.7 kg) compared to slow-growing animals (30.8 kg). The hot carcass weight was heavier (*P* ≤ 0.05) in the fast and intermediate-growing (24.57 and 19.07 kg) than slow-growing lambs (15.10 kg). The blood profiles of T3 and T4 hormones in addition to other parameters such as total protein, total lipids, and calcium level showed no clear variations among different experimental groups. At the molecular level, our data demonstrated upregulation of genes involved in protein biosynthesis (RPL7), fatty acid oxidation (CPT1), and lipolysis (FABP4) in the fast and intermediate-growing lambs in all studied tissues which facilitate protein accretion, energy expenditure, and fatty acid partitioning required for muscle building up. Moreover, the expression profile of the gene involved in muscle development (CAPN3) was increased in fast and intermediate-growing compared to slow-growing lambs in order to support muscle proper development. On the other hand, a candidate gene involved in lipogenesis (ADIPOQ) was expressed similarly in fat and liver tissues; however, its expression was increased in muscles of fast and intermediate-growing lambs compared to slow-growing animals. In conclusion, the current study indicated that the expression profile of genes involved in metabolic activities of liver, muscle, and adipose tissue is linked with the growth performance of lambs although no variations were detected in blood parameters. This provides an evidence for the importance of co-expression of these genes in body tissues to determine the final body weight and carcass characteristics of Barki sheep.

## Introduction

Sheep are considered one of the main sources of animal protein. In addition, sheep can survive in the desert by grazing low-quality forage (Elshazly and Youngs 2019). Barki sheep are raised under a transhumant animal farming system in the Northwestern coastal desert of Egypt with a population of 470,000 heads (11% of the total Egyptian sheep population). In addition, this breed is well-known to be well-adapted to the harsh Egyptian desert conditions (El-Wakil and Elsayed, 2013). Generally, individuals of the same sheep breed are varied in growth performance and final body weight. Therefore, selecting lambs that grow fast and obtain heavy final body weight is crucial for improving the meat production industry, which is the main target of sheep breeding programs (Parker et al. 1991; Moghaddam et al. 2021).

Indeed, investigation of factors that control sheep growth based on monitoring live body weights over a relevant time is of major interest among scientists and meat producers (Lupi et al. 2015; Moghaddam et al. 2021). The growth performance of farm animals is an important economic trait that regulated by genetic and non-genetic factors (Alemneh and Getabalew 2019). In addition, the growth performance of sheep is influenced by type of birth, sex, breed, season, age, and pre-mating weight of the dam, which all are considered non-genetic factors (Yilmaz et al. 2007). Moreover, factors such as slaughtering animals at an early age (immature body weight), poor genetic potential for growth, and inappropriate feeding plan are major factors that reduce meat production (Kefelegn et al., 2019). Therefore, it is required to get all information on growth rate and the degree of live body weight maturity relevant to carcass composition for a genetic selection plan (Massender et al. 2019). For example, lamb birth weight is a determinant trait that influences the survivability and meat production performance of the sheep farm (Buzanskas et al. 2014; Ptáček et al. 2017).

Determination of genetic factors controlling phenotypic variation of productive traits in domestic sheep (*Ovis aries*) will facilitate efforts done for accelerating genetic improvement. Moreover, the identification of genes underlying sheep growth performance would support worldwide efforts in increasing mutton production (Wang et al. 2015). In this regard, gene expression profile was linked with the growth performance of Chinese (Miao et al. 2015) and Egyptian sheep breeds (Ashour et al. 2020; Miao et al. 2015). However, the available data that linked genetic variants and growth performance and carcass traits of Egyptian sheep is still limited. Therefore, investigating the transcriptional profile of muscle tissues would provide useful information to improve the production and quality of sheep meat (Chao et al. 2016; Sun et al. 2016; Zhang et al. 2013a, b). Muscle growth and development are regulated by core genes and signal transduction pathways (Zhang et al. 2013a, b; Zhang et al. 2015). For example, single nucleotide polymorphism in RPL7 was associated with pre-weaning gain in mutton merino (Zhang et al. 2013a, b; Wang et al. 2015). Additionally, genes regulating fatty acid uptake and metabolism, such as FABP4 are considered candidates for meat tenderness (Xu et al. 2011). Therefore, the current research work aimed to study the relationship of gene expression profile in major body tissues (eye muscle, liver, and fat tail) with growth performance, carcass traits, and biochemical profile of Barki lambs.

## Materials and methods

### Experimental animals

The current study was conducted on Barki sheep flock, belongs to Maryout Research Station, 35 km south of Alexandria, Desert Research Center, Ministry of Agriculture and Land Reclamation, Egypt. Thirty-eight Barki lambs were kept and fed individually after weaning at age of 3 months (initial body weight) until 12 months of age (final body weight). The lambs were kept in a cubical cement box of dimensions (120 width*150 length*135 height cm) and have access to water and ration. Lambs were fed according to the standard schedule (NRC, 1985) which covers their nutritional requirements. Lambs were fed certain amounts of commercial concentrate mixture (12% crude protein) plus alfalfa hay (*Trifolium alexandrinum*). However, the amounts of concentrate feed mixture (CFM) offered to lambs were adjusted tri-weekly according to the live body weight. Moreover, water was available all the time for experimental animals.

### Experimental design

Lambs were weighed tri-weekly after weaning and divided into 3 groups according to their growth rates (fast, intermediate, and slow-growing). In addition, three samples of main body tissues (eye muscle, liver, and fat tail) from three animals representing each group were taken for gene expression profiling at the end of the fattening period (12 months of age). The genes which were selected for real-time PCR are regulators of different molecular pathways such as protein biosynthesis (RPL7), fat deposition or lipogenesis (ADIPOQ), fatty acid oxidation or lipolysis (CPT1 and FABP4), and muscle development (CAPN3).

### Slaughtering procedure and carcass trait

Twenty-three lambs were slaughtered at 12 months of age in the meat processing unit at Maryout Research Station to evaluate carcass traits according to the stranded protocol (Frild et al. 1961). Lambs were fasted for approximately 24 h before slaughter. After slaughtering and complete bleeding, the carcass was skinned and eviscerated before weighing. Weights of all non-carcass components (trachea, lungs, heart, liver, testes, spleen, kidneys, and kidney fat) were immediately weighed after removal from the body. The rumen and reticulum were cleaned and washed with cold running water. The carcasses were held in a chiller at 4 °C for 24 h to evaluate cold carcass weight (Frild et al. 1961).

### Blood and tissue sampling

Nine blood samples (3 from each group) were collected during slaughtering in tubes that contain EDTA as an anticoagulant substance. Tissue samples (liver, tail fat, and muscle) were taken immediately from lambs after slaughter and kept in RNA later until transferred into a − 80 °C freezer. The RNA extraction was performed in Cairo University Research Park, Faculty of Agriculture, Cairo University, Egypt.

### Analysis of blood T3 and T4 hormones profile

Blood samples were collected in tubes containing EDTA as an anticoagulant substance. Samples were centrifuged at 3000 rpm for 20 min. Plasma was stored at − 20 °C until estimation of T3 and T4 hormones using enzyme immunoassay test kits (Chemux Bioscience Inc, USA, CA). The intra and inter-assay variation coefficients were 5.0 and 13.0%, respectively.

### Measurement of blood total protein level

The profile total protein (g/dl) was done using the colorimetric method according to instructions provided by the manufacturer company (Bio diagnostic, Giza, Egypt). The plasma samples (0.025 ml) were mixed well with biuret reagent (1.0 ml) and incubated at 37 °C for 10 min before the absorbance for standard and samples were measured using the spectrophotometer at 550 nm wavelength.

### Measurement of blood glucose concentration

The blood glucose profile (mg/dl) was measured using the colorimetric method according to instructions provided by the manufacturer company (Bio diagnostic, Giza, Egypt).

### Measurement of blood total lipids level

The profile of blood total lipids (mg/dl) was measured using the colorimetric method according to instructions provided by the manufacturer company (Bio diagnostic, Giza, Egypt).

### Measurement of blood calcium level

The blood calcium level (mg/dl) was measured using the colorimetric method according to instructions provided by the manufacturer company (Bio diagnostic, Giza, Egypt).

### Gene expression profile

#### Total RNA extraction

The procedure of RNA isolation was performed using GeneJet RNA purification kit (Thermofisher Scientific, Vilnius, Lithuania) according to manufacturer instructions. Approximately 20 mg of tissue was weighed and grinded with a pestle in a mortar using liquid nitrogen till powder was formed. The powder was transferred into a 1.5 ml micro-centrifuge tube containing 300 μl of lysis buffer and 20 μl of β-mercaptoethanol and vortexed for 20 s. A volume of 600 μl of diluted Proteinase K (10 μl of Proteinase K diluted in 590 μl of Tris–EDTA (TE) buffer) was added and vortexed for 20 min. The samples were then incubated for 10 min at room temperature and finally centrifuged for 10 min at 12,000 × g. A volume of 450 μl of ethanol was added and mixed by pipetting. The lysate (700 μl) was transferred into the GeneJet RNA purification column and centrifuged for 1 min at 12,000 × g.

The flow through was discarded and the previous step was repeated until all the lysate was transferred and centrifuged. The collection tube was discarded, and the purification column was placed into a new 2 ml collection tube. A volume of 700 μl of wash buffer 1 was added to the purification column and centrifuged for 1 min at 12,000 × g. The flow through was discarded and the purification column was placed back into the collection tube, then 250 μl of wash buffer 2 was added to the purification column and centrifuged at 12,000 × g for 2 min.

The collection tube containing the flow through solution was discarded and the purification column was transferred into a sterile 1.5 ml RNase free micro-centrifuge tube. Nuclease free water (100 μl) was added to the purification columns and centrifuged at 12,000 × g for 1 min. For DNA digestion, 1 ml of DNAse (Thermo Scientific, California, USA) was added to 9 μl of RNA sample and the mixture (10 μl) was incubated in a thermal cycler for 30 min at 37 °C. After that, 1 ml of EDTA was added to the mixture and incubated in a thermal cycler (Thermofisher Scientific, CA, USA) for 10 min at 65 °C. Finally, the purity and concentration of extracted RNA were measured at A260/280 nm ratio (1.9–2.1) using NanoDrop 2000C (Thermofisher Scientific, Wilmington, DE, USA). The samples were stored at − 80 °C freezer until cDNA synthesis.

#### cDNA synthesis

After adjusting the RNA concentration of all isolated RNA samples, synthesis of cDNA was done using revertAid First Strand cDNA Synthesis Kit (Thermofisher, USA). The reaction mix consisted of 1 μl of oligo (dT) 18 primer, 11 μl of the adjusted RNA, 4 μl of 5 × reaction buffer, and 2 μl of 10 mM dNTP for each RNA sample. Finally, 1 μl of RiboLock RNase inhibitor and 1 μl of revertAid reverse transcriptase were added to reach a final volume of 20 μl. The samples were incubated in a thermal cycler (Thermofisher Scientific, CA, USA) for 60 min at 42 °C followed by 70 °C for 5 min. The cDNA samples were stored in − 20 °C freezer till used for real-time PCR runs.

#### Quantitative real-time PCR

A pair of primers (forward and reverse) was designed for each specific gene (GAPDH, ADIPOQ, CPT1, FABP4, RPL7, and CAPN3) using Primer3 software (http://primer3.wi.mit.edu//) as shown in Table 1. The design of primers was based on gene sequences described in the GenBank database (www.ncbi.nlm.nih.gov). Real-time PCR was performed using glyceraldehyde 3-phosphate dehydrogenase (GAPDH) as a housekeeping gene. The real-time PCR was done in 96 well plates (Thermofisher Scientific, Wilmington, DE, USA). The real-time PCR reaction mix is composed of 2 μl of cDNA sample, 12 μl of Maxima SYBR Green/ROX qPCR Master Mix (Thermofisher Scientific, CA, USA), 0.2 μl of specific reverse primer, 0.2 μl of specific forward primer, and 7.6 μl of nuclease free water. The reaction was incubated in StepOnePlus™ Real-Time PCR (Applied Biosystems, CA, USA). The reaction mix was incubated at 50 °C for 2 min; initial denaturation was done for 10 min at 95 °C and 40 cycles at 95 °C (denaturation) for 15 min and finally at 60 °C for 1 min (annealing), at 95 °C for 15 s and then 60 °C for 1 min. The results were expressed as Ct values and the relative gene expression profile was estimated using delta Ct analysis (^2−^ΔΔCT method) according to our recent study (Ghanem et al. 2021).

**Table 1:** Primer sequences of genes used for quantitative real-time PCR

Gene name	Gene bank accession number	Primer sequence	Fragment size (bp)
CPT1	NM_001009259.1	F: 5′- TCACCACTACGACCCAGAGG-3′ R: 5′- AGGACTTGTCGAACCACCTG-3′	95
ADIPOQ	KM216385.1	F: 5′- TTCCCATTCGCTTTACCAAG-3′ R:5′- CAAGTAGACGGTAATGTGGT-3′	122
FABP4	NM_001114667.1	F: 5′- GCCAGGAATTTGATGAAGTC-3′ R: 5′- ATTTCCCATCCCAGTTTTGT-3′	102
CAPN3	NM_001009212.1	F: 5′- GCCGCAATTTTCCCATTATT-3′ R: 5′- GTAAAACAGGGAGGTCTCG-3′	125
RPL7	XM_004011739.4	F: 5′- AAGCGACTGAGAAAGAAGTT-3′ R: 5′- CTGATGACAAACGCCAATTT-3′	191
GAPDH	NM_001034034.2	F: 5′- AGGTCGGAGTGAACGGATTC -3′ R: 5′- GGAAGATGGTGATGGCCTTT -3′	219

*PCR*, polymerase chain reaction; *bp*, base pair

#### Statistical analysis

Growth performance, carcass traits, biochemical measurements, and gene expression profile of selected candidate transcripts data were analyzed as a one-way analysis of variance using the SAS software, general linear model (v. 9.3, SAS Inst. Inc., Cary, NC, USA, 2011) as done by our group (Ghanem et al. 2014, 2021). The main effect was growth rate groups (fast, intermediate, and slow-growing animals). The following model was used: Y_ij_ = μ + G_i_ + e_ij,_ where:

- Y_ij_: The j^th^ observation of the i^th^ growth rate groups.
- μ: The overall mean.
- G_i_: The effect of the i^th^ growth rate groups (fast, intermediate, and slow-growing animals).
- E_ij_: Random error.

All data are reported as least square means (LSM) ± standard errors (SE). Mean values were separated when significance existed; using Duncan’s multiple range test (Duncan’s, 1955). The significance level was set at 5%.

## Results

### Growth performance traits

**Fig. 1:**
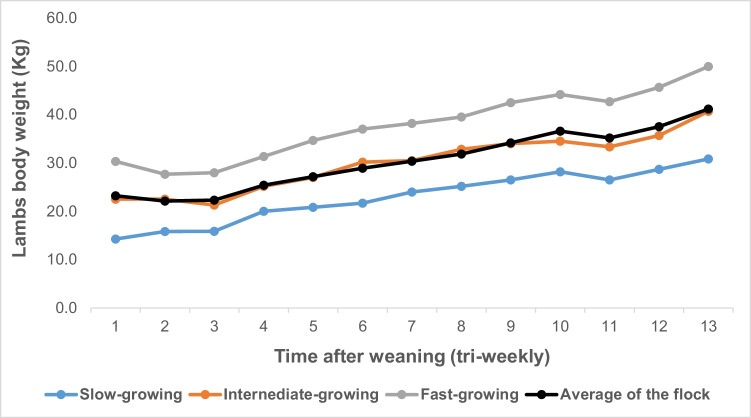
Growth rate of Barki lamb varied in growth performance under individual feeding management system

The birth weight of the fast-growing animals was similar to that of slow and intermediate-growing lambs. The least square means of initial body weight, the final body weight, average daily gain, and total body gain are shown in Table 2 and Fig. 1. The initial and final body weights were heavier (*P* ≤ 0.05) in the fast-growing than intermediate and slow-growing lambs. In addition, the initial and final body weights were greater (*P* ≤ 0.05) in the intermediate than slow-growing animals as shown in Table 2 and Fig. 1. Subsequently, the average daily gain was increased (*P* ≤ 0.05) in the fast and intermediate-growing compared to slow-growing animals. Additionally, the total body gain was (*P* ≤ 0.05) greater in the fast-growing than slow-growing animals.

**Table 2:** Growth performance traits of Barki lambs under individually feeding and management system

Growth parameters	Slow growing (mean ± S.E.)	Intermediate growing (mean ± S.E.)	Fast growing (mean ± S.E.)	Overall means	*R*^2^	C.V	*P*-value
Birth weight (kg)	3.83 ± 0.2	3.82 ± 0.2	4.10 ± 0.2	3.92	0.19	8.02	0.5249
Initial body weight (kg)	15.8 ± 1.2^**c**^	22.5 ± 1.2^**b**^	27.7 ± 1.2^**a**^	23.27	0.88	9.79	0.0016
Final body weight (kg)	30.8 ± 1.8^**c**^	40.7 ± 1.8^**b**^	49.9 ± 1.8^**a**^	40.85	0.90	7.93	0.001
Average daily gain (g)	92.3 ± 7.4^**b**^	112.0 ± 7.4^**ab**^	173.6 ± 7.4^**a**^	118	0.75	11.37	0.0155
Total body gain (kg)	15.0 ± 1.2^**b**^	18.2 ± 1.2^**ab**^	22.3 ± 1.2^**a**^	19.2	0.75	11.35	0.0154

Letters with the different superscripts in the same row were considered statistically significant at *P* ≤ 0.05. Data are expressed as mean ± standard error. *R*^2^ determination coefficient and *C.V*. coefficient of variation

### Carcass traits

The hot carcass weight (Table 3) was increased (*P* ≤ 0.05) in fast and intermediate compared to slow-growing lambs. Moreover, the liver weight of slaughtered lambs (Table 3) was significantly increased (*P* ≤ 0.05) in fast compared to intermediate and slow-growing lambs. On the other hand, dressing percentage, non-carcass fat, and tail fat were not significantly different among fast, intermediate, and slow-growing lambs. Furthermore, total body fat (non-carcass fat + tail fat kg) was increased but not significantly in fast and intermediate-growing compared to slow-growing lambs.

**Table 3:** Carcass traits of Barki lambs under individually feeding and management system

Item	Slow growing (mean ± S.E.)	Intermediate growing (mean ± S.E.)	Fast growing (mean ± S.E.)	Overall means	*R*^2^	C.V	*P*-value
Hot carcass weight (kg)	15.10 ± 1.0^**c**^	19.07 ± 1.0^**b**^	24.57 ± 1.0^**a**^	19.46	0.88	8.99	0.0018
Dressing percentage (%)	46.90 ± 0.3	47.48 ± 0.3	48.83 ± 0.3	47.70	0.35	2.74	0.2727
Non-carcass fat (%)	1.18 ± 0.8	1.21 ± 0.8	1.46 ± 0.8	1.22	0.11	34.79	0.7159
Liver (kg)	0.54 ± 0.2^**ab**^	0.50 ± 0.2^**b**^	0.62 ± 0.2^**a**^	0.55	0.70	6.12	0.027
Tail fat (%)	1.48 ± 0.8	1.790.34	1.650.34	1.74	0.07	35.99	0.793
Total body fat (%)	2.23 ± 0.6	2.61 ± 0.1	2.67 ± 0.4	2.57	0.07	29.80	0.7938

Letters with the different superscripts in the same row were considered statistically significant at *P* ≤ 0.05. Data are expressed as mean ± standard error. *R*^2^ determination coefficient and *C.V*. coefficient of variation

### The blood profile of T3 and T4 hormone

The blood profile of thyroid hormones (T3 and T4) were not significantly (*P* ≤ 0.05) different among fast, intermediate, and slow-growing lambs (Table 4).

**Table 4:** Thyroid hormones (T3 and T4), total protein, total lipids glucose, and calcium profile of Barki lambs under individually feeding and management system

Item	Slow growing (mean ± S.E.)	Intermediate growing (mean ± S.E.)	Fast growing (mean ± S.E.)	Overall means	*R*^2^	C.V	*P*-value
T3 (ng/ml)	1.0 ± 0.2	0.63 ± 0.2	1.10 ± 0.2	0.90	0.38	34.32	0.2363
T4 (ug/dl)	10.47 ± 2.1	12.37 ± 2.1	8.10 ± 2.1	8.49	0.25	35.69	0.4238
Total protein (g/dl)	6.07 ± 0.8	5.97 ± 0.2	6.53 ± 0.2	6.22	0.12	12.99	0.6724
Total Lipids (mg/dl)	392 ± 11.5	358 ± 48.8	378 ± 16.4	390	0.13	14.03	0.6499
Glucose (mg/dl)	74.17 ± 0.8	75.73 ± 0.6	74.87 ± 2.3	72.29	0.13	3.42	0.6496
Calcium (mg/dl)	10.87 ± 2.2	10.0 ± 1.3	10.13 ± 1.3	11.42	0.03	27.69	0.9243

Letters with the different superscripts in the same row were considered statistically significant at *P* ≤ 0.05. Data are expressed as mean ± standard error. *R*^2^ determination coefficient and *C.V.* coefficient of variation

### The blood profile of total protein

The total protein profile was not significantly different in fast, intermediate, and slow-growing lambs (Table 4).

### The blood profile of total lipids

The level of total lipids was not significantly different in fast, intermediate, and slow-growing lambs (Table 4).

### The blood profile of glucose level

The profile of blood glucose was not significantly different in fast, intermediate, and slow-growing lambs (Table 4).

### The blood profile of calcium profile

The concentration of blood calcium was not significantly different in fast, intermediate, and slow-growing lambs (Table 4).

### Gene expression profile of selected candidate transcripts

**Fig. 2:**
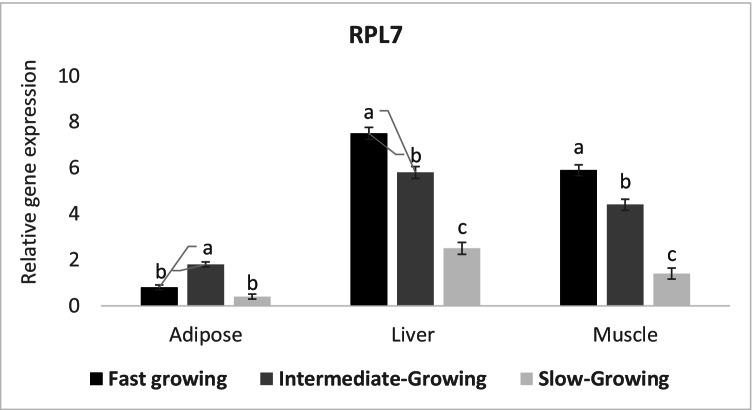
Expression profile of protein biosynthesis gene (RPL7) in different body tissues of Barki lambs varied in growth performance under individually feeding and management system

The expression profile of protein biosynthesis regulating candidate gene (RPL7) was significantly increased (*P* ≤ 0.05) in muscle (*Longissmus dorsi*) and liver tissues of fast and intermediate-growing compared to slow-growing lambs. On the other hand, adipose tissue of intermediate-growing animals recorded a higher expression profile of RPL7 gene than fast and slow-growing lambs (Fig. 2).

**Fig. 3:**
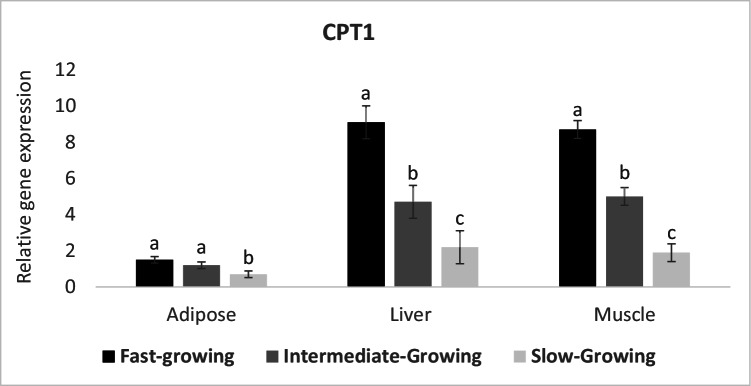
Expression profile of lipolysis gene (CPT1) in different body tissues of Barki lambs varied in growth performance under individually feeding and management system

The transcript abundance of CPT1 which is involved in β-oxidation process was increased significantly in muscle and liver samples of fast-growing lambs than intermediate and slow-growing animals. In addition, the intermediate-growing lambs had greater transcript abundance than slow-growing group (Fig. 3). However, the expression of this gene in fat tissue was increased (*P* ≤ 0.05) in fast and intermediate-growing than slow-growing lambs.

**Fig. 4:**
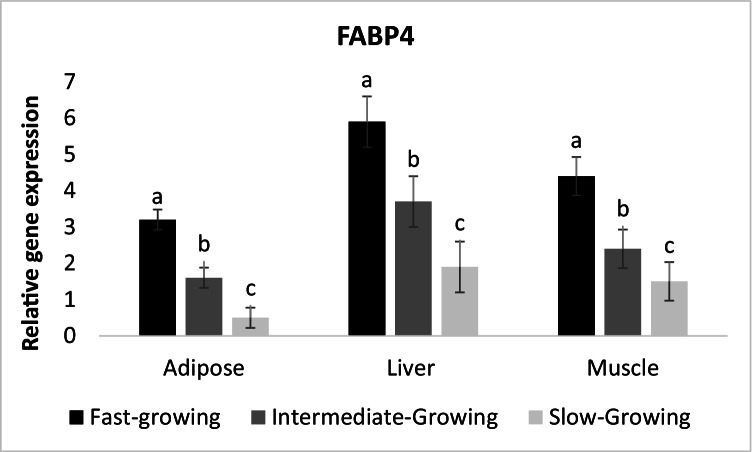
Expression profile of lipogenesis gene (FABP4) in different body tissues of Barki lambs varied in growth performance under individually feeding and management system

Noteworthy, transcript abundance of FABP4 (lipogenesis) was increased in all body tissues in fast and intermediate-growing compared with slow-growing lambs (Fig. 4).

**Fig. 5:**
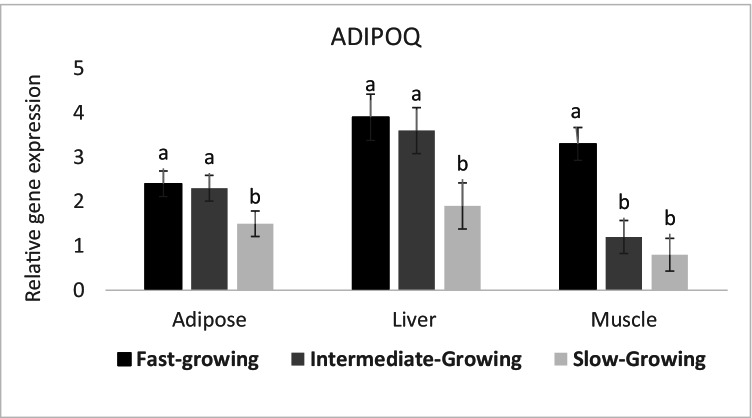
Expression profile of lipogenesis gene (ADIPOQ) in different body tissues of Barki lambs varied in growth performance under individually feeding and management system

The expression of ADIPOQ was increased (*P* ≤ 0.05) in muscle, liver, and fat tissues of fast and intermediate-growing compared to slow-growing lambs (Fig. 5). In addition, the adiponectin expression was higher in muscle collected from fast-growing than intermediate-growing lambs.

**Fig. 6:**
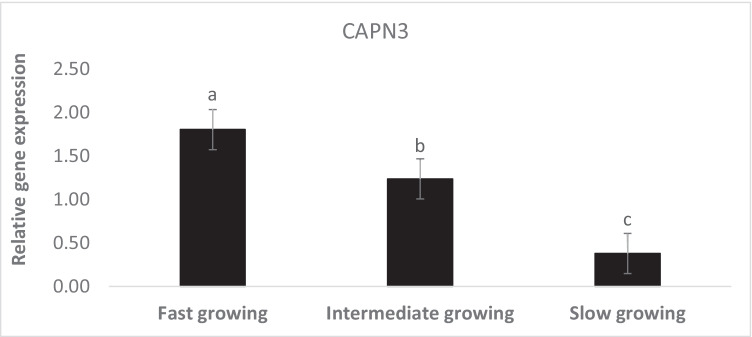
Expression profile CAPN3 in muscle of Barki lambs varied in growth performance under individually feeding and management system

The transcript abundance of CAPN3 was greater significantly in muscles of fast and intermediate than slow-growing animals (Fig. 6).

## Discussion

Growth traits such as birth weight, growth rate, body weight at slaughtering, and carcass characteristics of Barki sheep are crucial economic parameters. These parameters determine the productive efficiency of the animals to support the increased demand for mutton in Egypt (Sallam et al. 2012). Therefore, it is a privilege to select animals for meat production that grow fast and reached heavy bodies at slaughtering (Parker et al. 1991; Richards and Atkins 2004; Wang et al. 2015; Zhang et al. 2013a, b). The growth rate prior to weaning is considered a vital factor in determination of the production system performance. The fast-growing lambs, that quickly reach slaughter weight, will proportionately reduce the maintenance cost and increased farm revenue (Richards and Atkins 2004; Buzanskas et al. 2014; Wang et al. 2015; Zhang et al. 2013a, b).

Barki sheep is an Egyptian breed that survives well under the desert ecosystem, in addition to producing meat of high demand (El-Wakil and Elsayed 2013). The flock of Barki sheep of this investigation was under breeding program since 19,630 aiming to improve meat production (El-Wakil and Elsayed 2013). In the current study, the initial body weight of fast-growing lambs was greater than those intermediate and slow-growing lambs, which were positively linked with increased final body weight in fast-growing lambs. The total and average daily body gain were increased (*P* ≤ 0.05) in fast-growing lambs (22.3 kg and 173.6 gm) compared to intermediate (18.2 kg and 112.0 gm) and slow-growing lambs (15.0 kg and 92.3 gm). On the other hand, the growth rate of Awassi lambs was increased compared to Washera and Wollo highland breeds reflecting the genetic potentiality of body weight gain among these three breeds (Moghaddam et al. 2021).

Our results demonstrated that the final body weight of fast-growing animals was increased (49.9 kg) compared to intermediate (40.7 kg) and slow-growing groups (30.8 kg). The average body weights of Egyptian breeds were approximately 51 kg, 53 kg, and 44 kg at marketing age for Ossimi, Rahmani, and Barki breeds (Almahdy et al. 2000); Ashour et al. 2020; El-Malky et al. 2019; Hassan 2017; Khalifa et al. 2013; Marai et al. 2009; Miao et al. 2015). However, the body weights of Rahmani and Ossimi lambs were 40.8 and 36.6 kg at slaughtering under the same production system (Barkawi et al. 2009). In addition, daily weight gain and carcass traits of Awassi crossbred with Romanov were similar to that of pure Awassi breed (Tekel et al. 2020). On the other hand, Shaker et al. (2002) demonstrated that daily and total weight gain were increased in F1 lambs produced by crossbreeding Awassi ewes with Charollais and Romanov rams than the pure Awassi lambs. Indeed, individuals that grow fast with larger body size have greater economic returns; however, selection for lamb growth traits has been positively correlated with increased ewe mature body size (Borg et al., 2009; Posbergh and Huson 2021).

Birth weight of lambs is a key factor that influences growth performance of sheep (Buzanskas et al. 2014; Ptáček et al. 2017). The lamb final body weight is controlled by many genes such as MSTN, IGF-I, ADRB3, and leptin (Gholibeikifard et al, 2013). Within the muscle fibers, the biogenesis of ribosome and translation of protein are essential processes for the cells’ growth, differentiation, proliferation, and shaping animal development (Zhou et al. 2015). The building of muscle fibers and synthesis of protein required for animal growth are regulated partially by activity of ribosomal genes when proper nutrition is provided (Nader 2014). Indeed, the synthesis rate of muscle protein is mainly dependent on the content of ribosomes in muscle fibers (Millward et al. 1973). The expression profile of protein biosynthesis candidate gene (RPL7) was greater significantly in muscle (*Longissmus dorsi*) and liver tissues of fast and intermediate growing lambs than in slow-growing lambs which was in line with the increased body mass of fast and intermediate-growing lambs. Indeed, ribosomal proteins have a direct regulatory effect on the synthesis of protein that builds animal skeletal muscles (Costa et al. 2004; Han and Hickey 2005; Wang et al 2012). Protein is a crucial macronutrient participating in structural muscle building up in addition to its functional roles (hormones and enzymes) in the animal body (Mousa et al. 2019).

Moreover, it was found that the expression of RPL7 gene was downregulated in the broiler’s liver during feed restriction while there was no detection of differential expression of this gene in the adipose tissue (Wang et al. 2012). It was also reported that the genes involved in protein synthesis are altered severely due to diet restriction in porcine skeletal muscle (Costa et al. 2004; Han and Hickey 2005). Sheep genome-wide study proposed RPL7 as a candidate gene associated with growth performance and meat production traits (Zhang et al. 2013a, b). Additionally, RPL7 was linked with pre-weaning gain in German mutton merino (Wang et al. 2015). Noteworthy, our data indicated a higher expression profile of this gene in muscle and liver compared to adipose tissue. Thorrez et al. (2008) have demonstrated that the expression of RPL3 was enhanced in human adipose and muscle compared to liver cells. This spatiotemporal variation in gene expression between different species and tissues reflects changes of the cellular content of ribosomes and the activity of protein biosynthesis during different phases of body growth. In support of this idea, Cassar-Malek et al. (2007) indicated a change in the expression of ribosomal protein genes during different time points of bovine semitendinosus muscle development in the prenatal fetus stage.

Several studies have discovered genes associated with the post-weaning body weight of different sheep breeds (Gholizadeh et al. 2015; Al-Mamun et al. 2015). The upregulation of calpain3 (CAPN3) gene could be one of the main factors that enhanced the body mass of fast and intermediate-growing lambs more than the slow-growing animals. Calpains (CAPN) encode cysteine-activated intracellular proteases associated with increased number of myoblasts by controlling mitotic cycle (Barnoy et al. 1997; Cottin et al. 1994). Indeed, sheep individuals with TT genotype of CAPN had increased birth weight, final body weight, and average daily gain compared with other genotypes, while individuals with CC genotype recorded the lowest values of these traits (Mahrous et al. 2016). Several key transcripts involved in the development of skeletal muscles (MYL6B, MYH1, MYL3, MYO6, and MYOD1) were upregulated in the sheep fetal muscle that their maternal diet had high protein content (Sohel et al. 2020). This data indicates that increased dietary protein during pregnancy induces the expression of myogenic differentiation and development-related genes in the growing fetus.

The potential role of CAPN3 in enhancing sheep body weight has been reported by several authors who have demonstrated its genetic association with sheep growth performance and meat production traits (Koohmaraie 1992; Nassiry et al. 2007; Naveen et al. 2015). Therefore, it could be revealed that ribosomal and calpain encoded genes are co-expressed to enhance sheep body mass by stimulating myoblast proliferation and protein accumulation independent of the hormonal profile of thyroid hormones. Although increased plasma T4 concentration was recorded in Suffolk ewes compared to Gulf Coast native ewes, which was positively linked with larger body size and improved growth performance (Williams et al. 2004), however, our data did not show the same trend.

Animal body growth requires high-energy demand. In this regard, fatty acids are metabolized by an oxidation pathway in mitochondria to give acetyl-CoA, which is completely broken down via the Krebs cycle, or converted into acetate as an energy source (Guzman and Geelen 1993). Indeed, approximately 4–30% of circulating acetate and 10–55% of beta-hydroxybutyrate may be derived from hepatic oxidation of long-chain fatty acids in cattle (Mery et al. 1992). The catabolism of fatty acids occurs in the mitochondria by β-oxidation pathway (Sun et al. 2020). Our results demonstrated that the transcript abundance of CPT1 which is involved in the β-oxidation process was higher in muscle and liver samples of fast-growing lambs than intermediate and slow-growing lambs. Additionally, the intermediate-growing lambs had a greater transcript abundance of CPT1 than slow-growing lambs, while fast and intermediate-growing lambs had similar expression of this gene in fat tissue, although both had higher expression than slow-growing lambs.

A study that has been done in the fetal rabbit has shown increased expression of CPT I in cultured hepatocytes with long-chain fatty acids (Prip-Buus et al. 1995). The upregulation of this gene in fast-growing lambs may be due to increased metabolic demand to meet accelerated growth in fast and intermediate-growing lambs which is dependent on the utilization of fatty acids in both liver and developing muscle fibers. A second candidate of mitochondrial β-oxidation genes is known as CPT1B which was downregulated in the skeletal muscle of offspring that their maternal dietary nutrition was restricted (Muroya et al. 2021). This may be due to reduced fatty acid metabolism as a result of decreased uptake of fatty acids. In support to the importance of β-oxidation genes, Price et al. (2003) reported a tenfold increase in the expression of mRNA encoding CPT1B in mammary gland cells of lactating compared to late pregnant and control ewes. In addition, downregulation of CPT1b reduced fatty acid metabolism and subsequently resulted in the obesity of rats (Warfel et al. 2017). Noteworthy, a putative SNP overlapping gene encoding mitochondrial gene known as ATP5F1A was related to progeny birth weight and litter means weight at birth of Iranian Baluchi sheep (Esmaeili-Fard et al. 2021).

Liu et al. (2021) detected a higher transcriptional profile of ADIPOQ and FABP4 in the subcutaneous fat (SCF) of Holstein than Korean Wagyu-cross steers, suggesting that there were more active adipocytes in the SCF of Holstein steers. Indeed, the expression of both FABP4 and ADIPOQ genes was upregulated during differentiation of adipocytes which indicated that adipocytes maturity in Wagyu-cross steers was more advanced than that in Holstein steers (Albrecht et al. 2011; Liu et al. 2021). Bionaz et al. (2012) indicated that Madin–Darby bovine kidney cells increased oxidation of long-chain fatty acid through upregulation of FABP4 gene in the cytoplasm and CPT1A in the mitochondria. Our data indicated a higher transcript abundance of FABP4 in all examined body tissues in fast and intermediate growing than slow-growing lambs. A genetic SNP in FABP4 gene was associated with meat tenderness in sheep (Xu et al. 2011). The data of this study strongly support the idea that oxidation of long-chain fatty acid is a key determining molecular mechanism explaining the variation of sheep growth performance. Additionally, the transcript abundance of ADIPOQ was higher in muscle, liver, and fat tissues of fast and intermediate growing than slow-growing lambs. Moreover, the adiponectin expression level in muscle collected from fast-growing was higher than intermediated growing lambs.

Adipokines have an important biological role in regulating lipid metabolism (Reynolds and Vickers 2019). The transcript abundance of ADIPOQ was higher in muscle, liver, and fat tissues of fast and intermediate growing than slow-growing lambs. In addition, the adiponectin expression level in muscle collected from fast-growing was higher than intermediated growing lambs. It was demonstrated that expression of ADIPOQ in fat collected 1 week after calving had a positive correlation with free fatty acid and subsequently the mobilization of adipose body reserves (Elis et al. 2013). In a recent study, a genetic association between the growth and carcass traits and the ADIPOQ haplotypes was detected on New Zealand Romney lambs (An et al. 2017). Similarly, the fast-growing and intermediate growing sheep had higher expression of the ADIPOQ gene which was linked with their growth performance. Moreover, a genomic variant (SNP) in ADIPOQ was associated with the marbling score of Hanwoo cattle (Shin and Chung 2013; Choi et al. 2015). Therefore, this study suggested that ADIPOQ gene could be used to differentiate lambs that varied in growth performance and carcass traits in addition to its role in regulating fat deposition and differentiation.

## Conclusions

The data of the current investigation indicated that fast-growing lambs require upregulation of genes involved in protein biosynthesis (RPL7) which subsequently induced expression of muscle building up related transcript (CAPN3). The high demand of energy supply during muscle building up is provided by active transport of fatty acids through stimulation of FABP4 and ADIPOQ transcription. Finally, the utilization of fatty acids is performed inside mitochondria through β-oxidation process which is orchestrated by the CPT I gene. The co-expression of these genes in main body tissues is linked with growth performance and carcass traits of Barki lambs, which could be induced by the genetic makeup. However, this hypothesis requires a large population of lambs for validation.

## Data Availability

The data and materials of this study will be available on request.
